# A Standing Low-frequency Vibration Exercise Device for Improving Balance in Community-dwelling Older Adults: A Single-blind Randomized Controlled Trial

**DOI:** 10.1298/ptr.E10192

**Published:** 2023-02-07

**Authors:** Shinichiro OKA, Yoshio TAKANO, Hiroo MATSUSE, Yoshihiko TAGAWA, Naoto SHIBA

**Affiliations:** ^1^Department of Physical Therapy, Faculty of Rehabilitation, Reiwa Health Sciences University, Japan; ^2^Department of Physical Therapy, School of Health Sciences at Fukuoka, International University of Health and Welfare, Japan; ^3^Department of Orthopedics, Kurume University School of Medicine, Japan

**Keywords:** Older adults, Standing low-frequency vibration exercise device, Standing balance

## Abstract

Objective: This study aimed to compare the effects of the standing low-frequency vibration exercise device (SLVED) and walking training on balance ability on an unstable surface in community-dwelling elderly people. Methods: Thirty-eight older adults were randomly allocated to the SLVED sessions: the intervention group (n = 19), and the walking sessions: the control group (n = 19). Each group session lasted 20 min and was performed twice a week for 12 weeks. Standing balance was assessed by the change in center-of-gravity sway of the participant standing on foam rubber with eyes open (EO) and eyes closed (EC). The primary outcome measures were the root mean square (RMS) values of the center of foot pressure in the mediolateral and anteroposterior directions and the RMS area. Secondary outcome measures were the results of the 10-m walking time test (10 MWT), five-times sit-to-stand (5T-STS) test, and timed up-and-go (TUG) test. Results: Analysis of variance showed a significant group × time interaction for the TUG test. Significant improvements were observed in Y-RMS for EO condition; RMS, X-RMS, Y-RMS, and RMS area for EC condition; and 10 MWT, 5T-STS test, and TUG test for the main effect of the time factor. Conclusion: SLVED for intervention in community-dwelling older adults showed a greater improvement than walking training in the TUG test. In addition, SLVED improved the Y-RMS for the EO condition on foam rubber; RMS, X-RMS, Y-RMS, and RMS area for the EC condition on foam rubber in standing balance; and the 10 MWT and 5T-STS test, suggesting that it has similar effects to walking training.

**F**alls can reduce activities of daily living and increase nursing care due to trauma, such as fractures in older adults^1)^. Therefore, preventing falls in the elderly is essential to extend life expectancy and maintain quality of life. Previous studies have reported that a program comprising both balance training and muscle-strengthening exercises is effective at preventing falls in the elderly^2,3)^. However, most balance exercises are static and dynamic balance tasks in which the sensory input and ground surface are altered. These programs lack specificity for active community-dwelling older adults and may lack efficacy in reducing and preventing falls. To achieve effective balance training, it is essential to set tasks with an appropriate level of difficulty based on the subject’s ability.

Progressive balance exercises to prevent falls in daily life include step and equipment training. Step training can contribute to falling prevention by performing “correct,” “fast,” and “directional” steps^4)^. In addition, step practice under unstable conditions has been developed^5)^. However, there is a trade-off between the difficulty of balance training and the risk of falling, and step training has a higher risk of falling than static or dynamic balance tasks. Safe and effective balance-training equipment has been developed to address these issues. In addition, vibration stimulation interventions have been applied to improve balance.

A systematic review reported that a focused vibration device could effectively decrease the risk of falls and improve balance in the elderly^6,7)^. It was also reported that direct vibratory stimulation helped standing balance in community-dwelling older adults with eyes closed (EC)^8)^. However, previous stimulation methods, such as local stimulation of the Achilles tendon to activate sensation and stimulation from the sole in the standing position, are difficult to apply in combination with walking. Recently, passive exercise devices have been designed to improve walking ability. One product employing this method is the standing low-frequency vibration exercise device (SLVED)^9)^. The primary characteristic of SLVED is that it focuses on the movement of the lower limbs during the walking motion and incorporates the composite motion of (1) rocking stimulus in the left–right direction to shift the center of pressure (COP) to the stance leg side; (2) back-and-forth movement stimulus in the opposite phase; and (3) plantar dorsiflexion motion of the ankle joint to kick the ground during the terminal stance. A prior study showed that balance training with SLVED increases walking speed and improves ankle plantar dorsiflexion muscle strength and one-legged stance with EC condition^9)^. Thus, although the effects of SLVED on the motor system have been verified, its effects on sensory strategies in postural control have not been investigated. The modified Clinical Test of Sensory Interaction and Balance measures body sway in four different conditions to assess the dominance of the visual, somatosensory, and vestibular senses in controlling standing posture^10)^. It has also been suggested that the Berg Balance Scale static standing on a hard floor and sit-to-stand is not suitable for assessing balance ability in older adults with high activity levels^11)^. In contrast, the percentage of those unable to perform foam rubber static standing for 30 s in the EC condition, where the floor surface is unstable, increases in those over 60 years of age, and cutoff values for fall risk have also been reported^12)^. Therefore, we consider that the static standing balance of foam rubber could be used to evaluate the balance ability in sensory strategies of active community-dwelling older adults.

The SLVED was developed as a balance-training device that allows the safe performance of movements that simulate walking. Therefore, this study aimed to compare the effects of SLVED and walking training on standing balance on an unstable surface in community-dwelling older adults with walking independence in a randomized controlled trial.

## Methods

### Study design

**Fig. 1.**
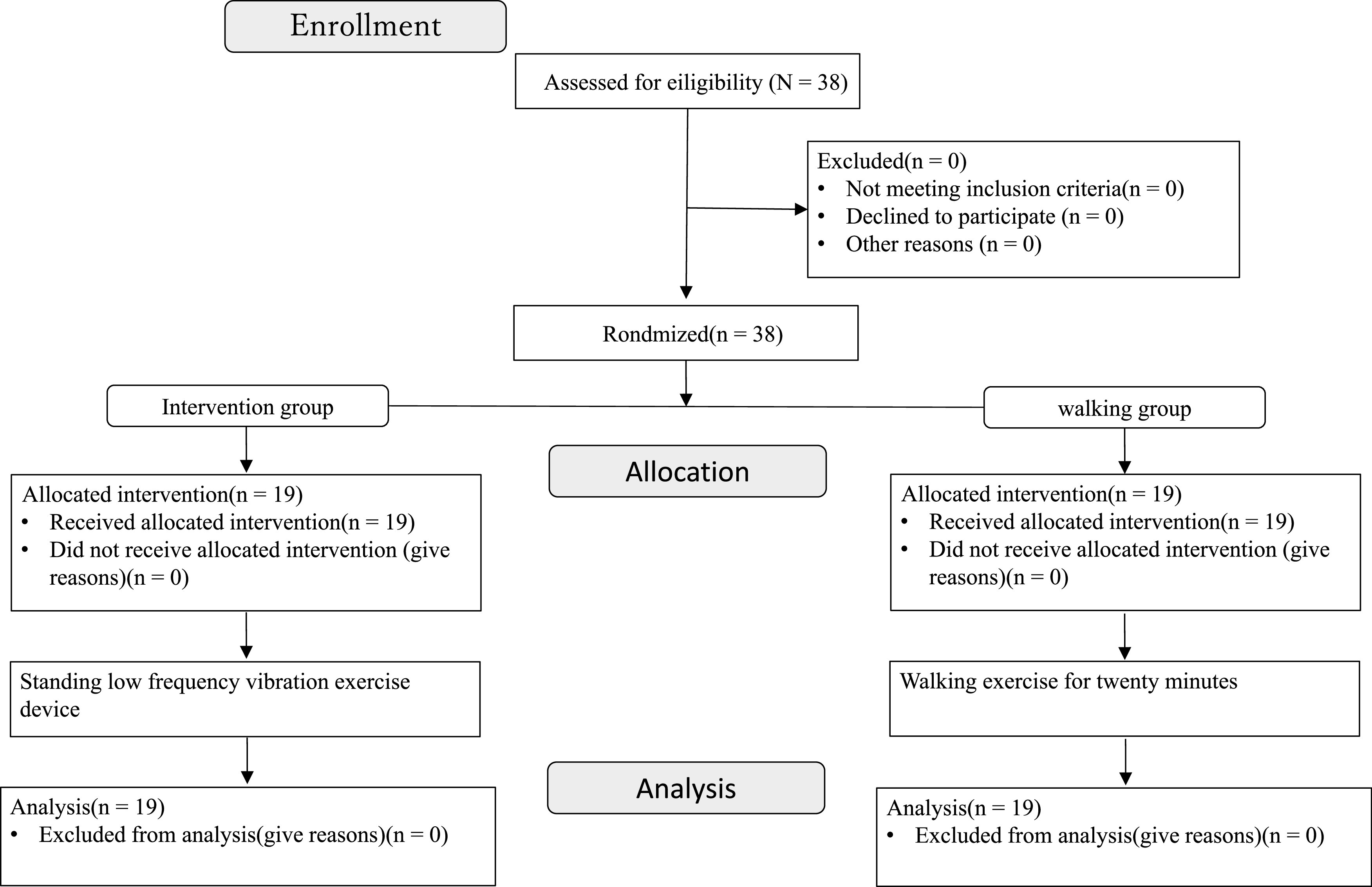
Flow diagram of the study

The study was designed as a 12-week single-blind randomized controlled trial with parallel arms, in which the raters were blinded. A flowchart describing the recruitment of participants and the study flow is presented in Figure 1. The participants were randomly assigned to SLVED sessions (intervention group) or walking sessions (control group) using a random number table created using the Rand function in Excel, and adjusted for sex and age. The assessments were conducted on day 1 (baseline and before session 1) and after 12 weeks, on day 7.

Participants were randomly allocated to the intervention group (SLVED) or walking group (walking exercise for twenty minutes).

SLVED, standing low-frequency vibration exercise device

### Participants

We enrolled 38 mentally capable people aged 65 years and older who could walk independently without the use of assistive aids. The exclusion criteria were as follows: history of neurological disease or orthopedic disease of the trunk and lower limbs, movement disorders, or diseases limiting their activities. Demographic characteristics included age, height, weight, body mass index, and Hasegawa Dementia Scale-Revised (HDS-R).

Participants provided written informed consent after receiving an explanation of the study. The study was begun after obtaining approval from the ethical review committee of International University of Health and Welfare to which we belong (approval number: 19-Ifh-088).

### Outcome measures

The primary outcomes to measure an improvement in the standing balance of participants with eyes open (EO) and EC conditions were the root mean square (RMS) amplitude of COP trajectories over time, in both the mediolateral (COP-X) direction for calculating X-RMS and anteroposterior (COP-Y) direction for calculating Y-RMS. The RMS area was also calculated.

The Twingravicoder G-6100 (ANIMA, Tokyo, Japan) and foam rubber CGT balance pad (IP-B6000, size: 540 mm × 478 mm × 74 mm; Inter Reha, Tokyo, Japan) were used to measure standing balance. These devices were used to measure the change in the degree of COP sway of the participant standing on the foam rubber under EO and EC conditions. The system recorded COP trajectories over time in both the mediolateral (COP-X) and anteroposterior (COP-Y) directions, at a sampling frequency of 20 Hz for 1 minute. One measurement was taken for each EO and EC condition. Participants stood on the foam rubber with both feet together. For the EO condition test, a target diameter of approximately 2 cm was placed 2 m in front of the subject at the eye level. A researcher instructed each participant to stand as still as possible while looking forward and keeping their arms relaxed at their sides. Participants were instructed to focus on the target as the recordings were taken. The steps for the EC condition test were the same, but without viewing the target. The RMS value (cm) of the amplitude was calculated for the COP, the X-RMS value (cm) of the amplitude was calculated for COP-X, and the Y-RMS value (cm) of the amplitude was calculated for COP-Y. The RMS area (cm^2^) was calculated as the area of a circle (π × RMS^2^), with the RMS value as the radius.

Secondary outcome measures were the 10-m walking time test (10 MWT), five-times sit-to-stand (5T-STS) test, and timed up-and-go (TUG) test. During the 10 MWT, a stopwatch was used to measure time while the participants walked at the maximum speed. The maximum 10 MWT was measured twice, and the best of the two results was used^13)^. The 5T-STS test is used as an index of lower-limb muscle strength in the elderly^14,15)^. In this test, the time taken for the participant to perform five STS repetitions on a standardized armless chair (0.40 m height) is measured. The participants were instructed to perform STS repetitions as rapidly as possible from a chair sitting position to a full standing position, with arms crossed over the chest. For the STS test, a stopwatch was used to measure the time until the participants sat down in their chairs after the fifth stand-up session to the nearest 0.01 s.

To measure TUG, the participant stood up from the armchair, walked 3 m, turned around, walked again, and recorded the time it took to sit down using a stopwatch^16)^. The TUG test was performed twice, and the best of the two results was used.

### Intervention

**Fig. 2.**
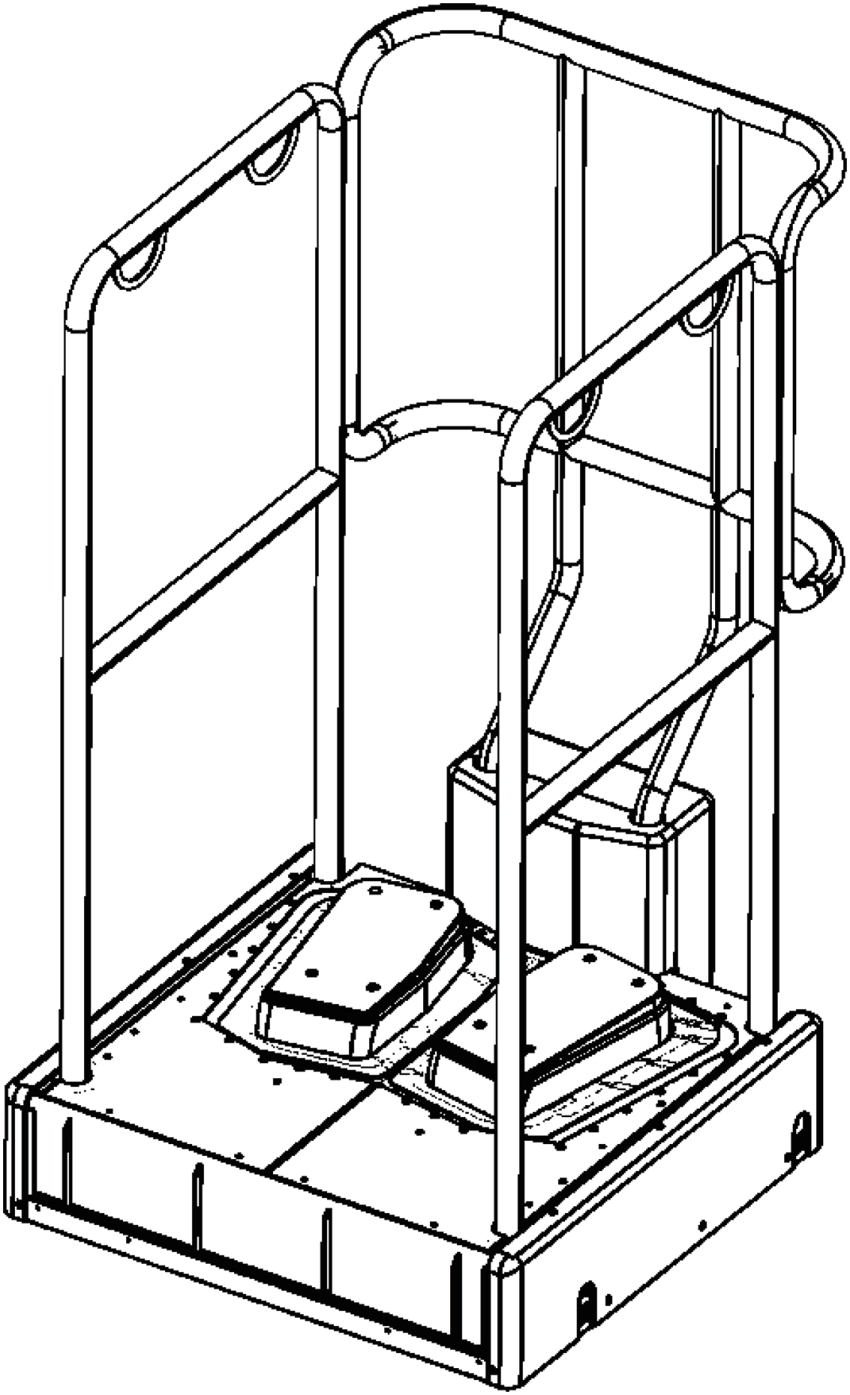
Original standing low-frequency vibration exercise device

A physiotherapist monitored both groups twice a week for 12 weeks. Participants in the intervention group stood on the SLVED for 15 minutes, for two sessions a week, totaling 24 sessions, while participants in the control group walked at a fast pace for 20 minutes, for two sessions a week, for a total of 24 sessions. A physiotherapist monitored both groups during the intervention. The SLVED 3D system Rakuraku Balance (Cotoho, Osaka, Japan) was set to shake participants at a frequency of 1.6 Hz (Fig. 2). The distance between the left and right footplates was 25 cm and the foot angle was 10°. The range of motion of the step on which the feet were placed was 10 mm forward and backward, 38 mm right and left, 5.5° plantar flexion, and 10.5° dorsiflexion. The footplate movement consisted of backward translation and dorsiflexion while shifting the center of gravity in the left and right movements, and forward translation and plantar flexion on the opposite side, which was performed in the reverse phase on the left and right sides. Subjects were instructed to maintain a standing position on their left and right footplates.

### Statistical analysis

Statistical analyses were performed using SPSS Statistics (version 25.0; IBM, Armonk, NY, USA). A non-paired t-test was conducted to compare the demographic characteristics and baseline outcomes between the intervention and control groups. In addition, analysis of variance (ANOVA) with a split-plot design was performed for comparison before and after the intervention (time factor), comparison of the intervention groups (group factor), and interaction (time × group).

The effect sizes (eta squared, η_p_^2^) were calculated for the main and interaction effects. The eta-squared (η_p_^2^) value of the ANOVA tests indicated effect sizes following Cohen’s guidelines (0.01, small; 0.06, medium; 0.14, large)^17)^. A corresponding paired t-test was performed to compare outcomes before and after the intervention. Cohens’ d was used to measure the effect size, with values of 0.14, 0.4, and 0.75 indicating small, medium, and large effects^18)^. The significance level was set at 5%.

## Results

### Flow of participants through the trial

Thirty-eight people (8 males, 30 females; 77.6 ± 4.0 years) were screened, all of whom met the study criteria, signed an informed consent form, and were subsequently enrolled in the study. This cohort was split into the intervention and control groups (n = 19 each), and all participants completed the relevant intervention and measurement periods (Fig. 1).

The demographic characteristics (Table 1) and baseline values of the outcome measures (Tables 2 and 3) were comparable between the groups, except for the TUG and RMS area under the EC condition. In the baseline comparison, the RMS area under the EC condition of the intervention group was larger than that of the control group (p = 0.032). In addition, the TUG test of the control group was performed earlier than that of the intervention group (p = 0.010).

**Table 1.** Participants’ characteristics

	Intervention	Control	p-value
Number (male/female)	19 (4/15)	19 (4/15)	
Age, year	78.0 (3.8)	77.4 (3.8)	0.641
Height, m	1.54 (9.4)	1.53 (9.6)	0.907
Weight, kg	55.5 (8.2)	55.2 (9.7)	0.929
BMI, kg/m^2^	23.5 (3.1)	23.3 (3.8)	0.885
HDS-R	25.1 (2.8)	26.3 (2.8)	0.194
Average (standard deviation), non-paired t-test			
BMI, body mass index; HDS-R, Hasegawa Dementia Scale-Revised			

**Table 2.** Changes from pre and post for all groups of gravity sway test in EO and EC

	Intervention			Control			F-value					
	Pre	Post	d	Pre	Post	d	Time	η_p_^2^	Group	η_p_^2^	Time × Group	η_p_^2^
EO												
RMS, cm	1.38 (0.07)	1.32 (0.06)	0.23	1.34 (0.05)	1.31 (0.06)	0.11	2.135	0.056	0.101	0.003	0.328	0.009
X-RMS, cm	0.94 (0.05)	0.93 (0.05)	0.04	0.88 (0.04)	0.92 (0.05)	0.19	0.685	0.008	0.006	0.008	0.685	0.019
Y-RMS, cm	1.00 (0.06)	0.93 (0.05)	0.35	1.00 (0.05)	0.92 (0.05)	0.34	5.392^#^	0.130	0.006	0.000	0.019	0.001
RMS area, cm^2^	6.26 (0.70)	5.65 (0.54)	0.23	5.78 (0.45)	5.59 (0.54)	0.09	2.055	0.054	0.132	0.004	0.590	0.016
EC												
RMS, cm	2.26 (0.16)	1.94 (0.12)*	0.52	1.90 (0.07)	1.78 (0.09)	0.37	8.483^##^	0.191	3.471	0.088	1.475	0.039
X-RMS, cm	1.57 (0.11)	1.34 (0.08)*	0.55	1.31 (0.06)	1.20 (0.08)	0.35	7.532^##^	0.173	3.706	0.093	1.059	0.029
Y-RMS, cm	1.61 (0.12)	1.41 (0.09)*	0.44	1.37 (0.05)	1.30 (0.06)	0.34	7.078^#^	0.164	2.667	0.069	1.375	0.037
RMS area, cm^2^	17.38 (2.41)	12.67 (1.51)**	0.54	11.68 (0.83)^†^	10.33 (1.03)	0.33	6.656^#^	0.156	4.554^#^	0.112	2.653	0.054
Average (standard error), ANOVA: ^#^p <0.05, ^##^p <0.01												
Significant difference between pre and post (paired t-test): *p <0.05, **p <0.01												
Comparing groups in baseline (non-paired t-test): ^†^p <0.05												
Effect size (η_p_^2^), ANOVA (0.010: small, 0.060: medium, 0.140: large)												
Effect size (Cohen’s d), pre vs post (0.15: small, 0.40: medium, 0.75: large)												
EO, eyes open; EC, eyes closed; RMS, root mean square; ANOVA, analysis of variance												

### Primary outcomes: changes in standing balance on an unstable surface

We found no interaction between the time and group factors of the COP sway test for any items under either the EO or EC conditions. We found a significant time factor and main effect for Y-RMS (F (1, 36) = 5.392, p = 0.026, η_p_^2^ = 0.130) for participants in the EO condition, and RMS (F (1, 36) = 8.483, p = 0.006, η_p_^2^ = 0.191), X-RMS (F (1, 36) = 7.532, p = 0.009, η_p_^2^ = 0.173), Y-RMS (F (1, 36) = 7.078, p = 0.012, η_p_^2^ = 0.164), and RMS area (F (1, 36) = 6.656, p = 0.014, η_p_^2^ = 0.156) for participants in the EC condition. Furthermore, we observed a significant group factor and main effect for the RMS area for participants in the EC condition (F (1, 36) = 4.554, p = 0.040, η_p_^2^ = 0.112).

In the intervention group, RMS, X-RMS, Y-RMS, and the change in RMS area from baseline to the end of the exercise sessions for participants in the EC condition showed significant improvements, with a medium effect (RMS, p = 0.006, d = 0.52; X-RMS, p = 0.011, d = 0.55; Y-RMS, p = 0.010, d = 0.44; RMS area, p = 0.007, d = 0.54) (Table 2).

In the control group, there was no significant difference between the pre- and post-intervention COP sway tests under either the EO or EC conditions for any items.

### Secondary outcomes

#### 10 MWT

We found no interaction between the time and group factors in the 10 MWT. However, a large and significant time effect (F (1, 36) = 11.145, p = 0.002, η_p_^2^ = 0.236) was observed at 10 MWT. The post-hoc test also showed a statistically significant improvement in the intervention group, with a moderate effect (d = 0.50), but no significant difference in the control group (Table 3).

**Table 3.** Changes from pre and post for all groups of 10 MWT, 5T-STS test and TUG test

	Intervention			Control			F-value					
	Pre	Post	d	Pre	Post	d	Time	η_p_^2^	Group	η_p_^2^	Time × Group	η_p_^2^
10 MWT, s	5.6 (0.3)	5.1 (0.2)**	0.50	5.2 (0.1)	5.0 (0.1)	0.31	11.145^##^	0.236	1.771	0.047	1.485	0.040
5T-STS test, s	8.0 (0.4)	6.9 (0.4)**	0.57	7.3 (0.3)	6.3 (0.3)**	0.83	52.067^#^	0.591	1.492	0.040	0.138	0.004
TUG test, s	7.7 (1.6)	6.4 (0.7)**	1.01	6.6 (0.7)^†^	6.2 (0.7)	0.47	14.834^##^	0.292	6.861^#^	0.160	4.190^#^	0.104
Average (standard error), ANOVA: ^#^p <0.05, ^##^p <0.01												
Significant difference between pre and post (paired t-test): **p <0.01												
Comparing groups in baseline (non-paired t-test): ^†^p <0.05												
Effect size (η_p_^2^), ANOVA (0.010: small, 0.060: medium, 0.140: large)												
Effect size (Cohen’s d), pre vs post (0.15: small, 0.40: medium, 0.75: large)												
10 MWT, 10-m walking time test; 5T-STS, five-times sit-to-stand; TUG, timed up-and-go; ANOVA, analysis of variance												

#### 5T-STS test

We found no interaction between the time and group factors in the 5T-STS test. We found a significant time effect (F (1, 36) = 52.067, p <0.001, η_p_^2^ = 0.591) for the 5T-STS test. In addition, post-hoc testing revealed a statistically significant improvement over time, with a medium effect (d = 0.57) in the intervention group and a large effect (d = 0.83) in the control group (Table 3).

#### TUG test

We found a significant medium time × group interaction effect (F (1, 36) = 4.190, p = 0.048, η_p_^2^ = 0.104). Furthermore, a significant, large group and time effect (group: F (1, 36) = 6.861, p = 0.013, η_p_^2^ = 0.160; time: F (1, 36) = 14.834, p = 0.002, η_p_^2^ = 0.292) were also observed. Comparison of TUG before and after exercise showed a large effect on the improvement after 24 sessions of exercise in the intervention group (p <0.001, d = 1.01) but no significant difference in the control group (Table 3).

## Discussion

In this randomized controlled trial, we compared the effects of SLVED and walking training on standing balance in foam rubber, STS test, gait, and TUG test in community-dwelling older adults. ANOVAs revealed a significant group × time interaction for the TUG test. The main effect of the time factor was found in standing balance for both the EO and EC conditions on foam rubber, 10 MWT, 5T-STS test, and TUG test. In addition, the intervention group showed statistically significant improvements from baseline in RMS, X-RMS, Y-RMS, RMS area with EC, 10 MWT, 5T-STS test, and TUG test. These results suggest that SLVED has a similar effect to walking training in community-dwelling older adults.

We found that TUG test was observed for medium interaction effects, and the large-effect group factor and control group were faster than the intervention group at baseline. For participants tested under the EC condition, the RMS area showed a medium group factor effect, which was larger in the intervention group than in the control group at baseline. During the TUG test, participants stood up from a chair, walked, and performed 180-degree turn movements. There were no differences between the groups at baseline in the 10 MWT, which is a measure of walking ability, or the 5T-STS test, which is a measure of standing movement. Therefore, the two groups were considered to have similar walking and sit-to-stand abilities. However, there was a group difference in the RMS area under the EC condition, which is a measure of postural control by the vestibular system. These results suggest that this difference may influence the interaction of vestibular function in the TUG test during turning.

A main effect of time was observed on the foam rubber standing balance with EO and EC conditions, 10 MWT, 5T-STS test, and TUG test. In addition, the simple main effect in the intervention group showed statistically significant improved outcomes from medium to large effects for RMS, X-RMS, Y-RMS, and RMS area with the EC condition, 10 MWT, 5T-STS test, and TUG test. These results agree with those of a previous study that reported that vibratory stimuli applied to the plantar region exert beneficial effects on balance in women aged 60 years or older, with greater efficacy in anterior displacement, postural control of the anteroposterior axis, and EC condition^8)^. Standing balance with the EC condition on foam rubber modifies somatosensory input from the plantar surface and increases dependence on vestibular sensation^12,19)^. During postural control, the visual image is maintained by the vestibulo-ocular reflex (VOR), in which the vestibular senses receive head movements, and the eyes move at the same speed in opposite directions. VOR adapts in a speed-dependent manner at frequencies above 0.3 Hz, and VOR adaptation training has been reported to be more effective at medium frequencies (1.3 Hz)^20)^. A previous study reported that the average daily gait cycle of elderly Japanese individuals is 1 Hz^21)^. SLVED participants maintained a standing position on the footplate with EO during compound movements at a frequency of 1.6 Hz. This shows that SLVED with EO may elicit VOR to maintain the visual image and stimulate vestibular sensation in response to head movements linked to footplate movements. As a result of the stimulation, vestibular function to control head movements was improved, and body sway was reduced while participants stood on an unstable surface.

In both groups, the 5T-STS test had a simple main effect and significant improvement (intervention group: moderate effect, control group: large effect). Walking is known to improve knee extension torque^22)^, which likely explains these results. A previous study investigating locomotive syndrome in Japanese individuals reported a 5T-STS of 8.25 ± 2.23 s in healthy elderly participants^23)^. Tankisheva et al. reported that local vibration treatment of the mid thigh and around the hip for six months increased knee extension muscle torque in postmenopausal women^24)^. Bellomo et al. reported that global sensorimotor and high-intensity focused vibration for 8 weeks increased knee extension muscle torque in elderly individuals with sarcopenia^25)^. The 5T-STS test has been reported to be an indicator of knee extension muscle strength^14)^ and balance ability in patients with vestibular disorders^26)^. These results suggest that SLVED and walking training improved lower-extremity muscle strength and balance ability during standing and sitting in older adults.

Participants standing on foam rubber with EO condition were dependent on vision, which was the main effect observed in the Y-RMS, an index of body sway in the anterior-posterior direction that decreased after the intervention. In the device used in this study, the left–right inverse phase of the ankle plantar dorsiflexion and hip extension movements by foot support was similar to the lower limb movements during walking^9)^. Therefore, it is assumed that holding the standing position with the EO condition on the device adapts to the anterior-posterior movement of the body. A previous study reported that anterior–posterior body swaying appears in the standing position with visual stimuli in which the visual landscape flows back and forth^27)^. In addition, it has been reported that the relationship between the distance of the gazing index and body sway in the upright position increases with distance^28)^. Thus, anteroposterior body sway is affected by visual information. However, it has been reported that ankle plantar flexors and dorsiflexors contribute to ankle plantar flexion and dorsiflexion strategies, respectively^29,30)^. These results suggest that the main effects of ankle plantar dorsiflexion and visual stimulation in the anteroposterior direction were reduced in participants in the EO condition in the control and intervention groups. However, since there was no simple main effect before and after the intervention in each group, it was assumed that the effect was small in community-dwelling older adults.

## Conclusion

This study compared the effects of SLVED and walking training on foam rubber standing balance, 5T-STS test, 10 MWT, and TUG test in older adults living in the community. The results showed that SLVED had similar effects as walking training on foam rubber standing with EC condition, walking, 5T-STS test, and TUG test in participants. The specific SLVED protocol used in this study can be considered safe and suitable for mobility and balance training programs in community-dwelling older adults.

The limitations of this study include the bias in TUG test and balance assessment at baseline at random assignment and the lack of investigating vestibular function assessment as a factor. Further studies with larger sample sizes, taking into account allocation adjustment by the main outcome, and somatosensory and vestibular function assessments should be conducted to verify our findings. In addition, training time and frequency should be increased with a passive exercise-assisting device.

## Conflict of Interest

Yoshio Takano received research funding and materials from Cotoho Co., Ltd. The other authors have no conflicts of interest to declare.
